# Basosquamous carcinoma in a patient with Freeman-Sheldon syndrome

**DOI:** 10.1016/j.jdcr.2026.04.043

**Published:** 2026-04-29

**Authors:** Andrea Gispert, Eric Cline, John Scopetta

**Affiliations:** a Clear Choice Dermatology, Lake Oswego, Oregon; b Baptist Health, Transitional Year Program, Birmingham, Alabama; c Frontier Dermatology, Seattle, Washington

**Keywords:** basal cell carcinoma, basosquamous carcinoma, Freeman-Sheldon syndrome, metatypical basal cell carcinoma

## Introduction

Freeman-Sheldon syndrome (FSS) is a musculoskeletal condition that tends to involve the face, hands, feet, and is often traced to the myosin heavy chain 3 (*MYH3*) gene. Patients tend to have microstomia giving the appearance that they are whistling.^1^ Complications include feeding difficulties and dysphagia.^2^ From a surgical standpoint, management can be challenging because patients with FSS have been reported to develop malignant hyperthermia following exposure to general anesthesia.^3^

Basosquamous carcinoma (BSC) demonstrates mixed basal and squamous cell carcinoma features. BSC is believed to originate as a basal cell carcinoma (BCC) that ultimately undergoes genetic alterations to exhibit features of squamous differentiation. Clinically, BSC behaves aggressively with higher rates of postsurgical local recurrence.^4^

To date, only a limited number of cases of FSS exist in the literature, and most focus on the orthopedic and developmental consequences of the condition.^5^ No cases have ever been published about a patient with FSS having BSC at an early age. In this case report, we present a patient with FSS that developed a BSC in his 30’s.

## Case Report

**Fig 1:**
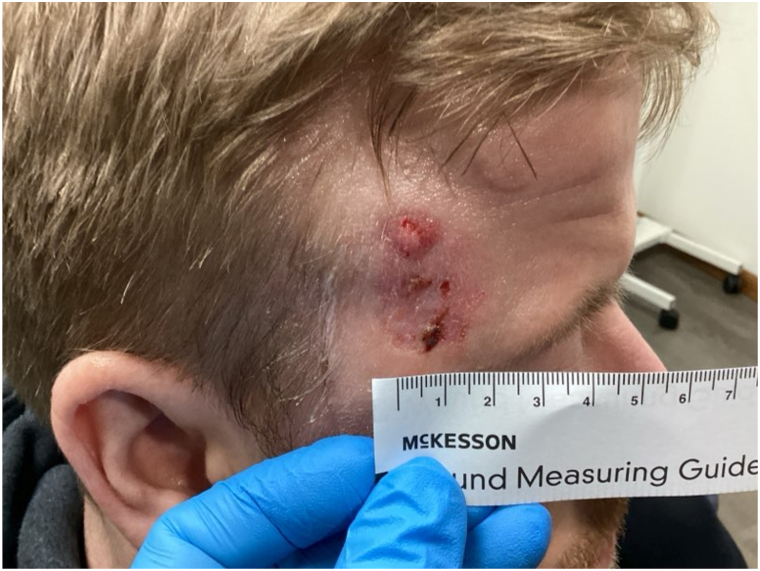
Pearly, erythematous plaque with central ulceration, hemorrhagic crust, and lateral spread on the right temple.

**Fig 2:**
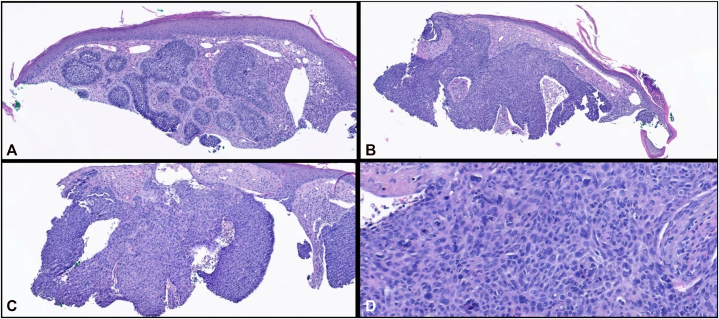
H&E stains of basosquamous carcinoma. **A,** 10× magnification: Section demonstrating typical nodular basal cell carcinoma morphology with peripheral palisading and fibromyxoid stroma. **B,** 5× magnification: Tumor nodules exhibiting both basal cell and squamous morphologies, with areas of larger, pleomorphic nuclei and eosinophilic cytoplasm. **C,** 10× magnification: Adjacent regions showing conventional basophilic basal cell carcinoma with clefting and fibromyxoid stroma alongside squamoid areas. **D,** 20× magnification: Higher-power view of squamoid regions, highlighting marked nuclear pleomorphism, cellular atypia, and eosinophilic cytoplasm.

A 34-year-old man with a past medical history of FSS, without a known family history of the condition, as well as glaucoma, malignant hyperthermia and autism spectrum disorder, presented for evaluation of an enlarging skin lesion on the right inferior temple present for the past 3 years. During his dermatology encounter, genetic confirmation of FSS was not clarified; however, the patient exhibited characteristic phenotypic features of FSS and had a prior history of malignant hyperthermia, a recognized association of FSS.^3^ Previously, he had been prescribed and treated with topical steroids and antibiotic ointment without any improvement. The patient endorsed the lesion as painful, red, nonhealing, and itchy. He denied any personal history of skin cancer, significant history of sun exposure, ionizing radiation exposure, immunosuppressive conditions or medications, atypical nevi, dysplastic nevi, and any familial history of skin cancer. On physical exam of the right temple, a pearly plaque with central ulceration, hemorrhagic crust and lateral spread was observed (Fig 1). Due to the nonhealing nature and concerning appearance, the lesion was biopsied and sent to pathology. The resultant pathology showed malignant dermal nodules and small nests of basaloid cells with peripheral palisading (Fig 2). Basaloid cells observed had increased nuclear to cytoplasmic ratios, increased mitoses and apoptoses. Admixed islands of pink, squamoid cells were also identified within the basaloid nodules. SOX10 staining showed only background epidermal melanocytes. The patient was diagnosed with BSC. Mohs surgery could not be performed due to the patient’s glaucoma pressure necessitating near constant head repositioning. The patient was referred to an academic center for wide-local excision under general anesthesia, as this syndrome is associated with known anesthetic complications.^3^ The postexcisional pathology report was negative for lymphovascular invasion and showed clear margins. The patient was advised to return to his primary dermatologist for skin cancer screenings every 6 months.

## Discussion

There are several known genetic syndromes that predispose individuals to BCC such as Gorlin syndrome, Brooke-Spiegler syndrome, xeroderma pigmentosum, and others.^6^ FSS has not been described as a cancer-predisposition syndrome, making the development of BSC in this patient unexpected. Generally, men below the age of 40 are not at significant risk of developing BCC. However, environmental exposures to radiation and asbestos may elevate one’s chances of acquiring BCC. In one study, 50 individuals below 40-years-old were age matched to 27 controls to determine what additional factors influenced BCC diagnoses in a younger cohort. There was statistical significance found in individuals with BCC based on outdoor occupation and family history of skin cancer.^7^ Notably, the patient in our case reported neither significant sun exposure nor a family history of skin cancer, making him an outlier among an already unusual population. This could suggest a possible biological or structural vulnerability of the skin in those with FSS.

One gene that has been identified in patients with FSS is the *MYH3* gene, which is responsible for embryonic skeletal muscle myosin heavy chain 3. However, some instances of FSS do not have any changes to this gene. While some cases of FSS have happened sporadically without any family history, in other cases, there has been an autosomal dominant and recessive pattern of inheritance.^1^ To date, there is no evidence that pathogenic *MYH3* variants, or other rare genetic causes of FSS like *TNNI2*, are associated with defects in DNA repair or immune surveillance; however, these factors alone do not fully account for cancer risk.^8^ The absence of documented genetic confirmation of FSS in this patient represents a limitation of this report. However, molecular testing is not always universally available or financially feasible, and the clinical features observed in this case nonetheless provide a valuable hypothesis-generating observation. Therefore, the molecular basis for the early development of BSC in this patient remains unclear, and further investigation would be required to determine whether any indirect or as-yet-unrecognized mechanisms may contribute. The complete absence of reported cases may also reflect the rarity of the syndrome and underdiagnoses of skin cancer in this population, especially in the absence of routine dermatologic surveillance.

## Conclusion

The occurrence of an aggressive BSC at an unusually early age in a patient with FSS is notable and, to our knowledge, has not been previously reported. While causality cannot be inferred from a single case, this observation raises the hypothesis that genetic alterations underlying FSS, including pathogenic variants in *MYH3*, could potentially modulate carcinogenic susceptibility through currently unrecognized mechanisms. Further investigation through additional case reports or genotype–phenotype correlation studies will be necessary to explore this possibility.

## Declaration of generative AI and AI-assisted technologies in the writing process

Artificial intelligence tools were not used in the writing, editing, or preparation of this manuscript. Artificial intelligence was not utilized in the generation, production, or manipulation of any images contained within this case report.

## Conflicts of interest

None disclosed
